# Quantitative Structure-Activity Relationship Modeling of the Amplex Ultrared Assay to Predict Thyroperoxidase Inhibitory Activity

**DOI:** 10.3389/fphar.2021.713037

**Published:** 2021-08-12

**Authors:** Domenico Gadaleta, Luca d’Alessandro, Marco Marzo, Emilio Benfenati, Alessandra Roncaglioni

**Affiliations:** Laboratory of Environmental Chemistry and Toxicology, Department of Environmental Health Sciences, Istituto di Ricerche Farmacologiche Mario Negri IRCCS, Milan, Italy

**Keywords:** thyroid, thyroperoxidase, TPO, endocrine disruptors (E.D.), QSAR, non-testing methods

## Abstract

The thyroid system plays a major role in the regulation of several physiological processes. The dysregulation of the thyroid system caused by the interference of xenobiotics and contaminants may bring to pathologies like hyper- and hypothyroidism and it has been recently correlated with adverse outcomes leading to cancer, obesity, diabetes and neurodevelopmental disorders. Thyroid disruption can occur at several levels. For example, the inhibition of thyroperoxidase (TPO) enzyme, which catalyses the synthesis of thyroid hormones, may cause dysfunctions related to hypothyroidism. The inhibition of the TPO enzyme can occur as a consequence of prolonged exposure to chemical compounds, for this reason it is of utmost importance to identify alternative methods to evaluate the large amount of pollutants and other chemicals that may pose a potential hazard to the human health. In this work, quantitative structure-activity relationship (QSAR) models to predict the TPO inhibitory potential of chemicals are presented. Models are developed by means of several machine learning and data selection approaches, and are based on data obtained *in vitro* with the Amplex UltraRed-thyroperoxidase (AUR-TPO) assay. Balancing methods and feature selection are applied during model development. Models are rigorously evaluated through internal and external validation. Based on validation results, two models based on Balanced Random Forest (BRF) and K-Nearest Neighbours (KNN) algorithms were selected for a further validation phase, that leads predictive performance (BA = 0.76–0.78 on external data) that is comparable with the reported experimental variability of the AUR-TPO assay (BA ∼0.70). Finally, a consensus between the two models was proposed (BA = 0.82). Based on the predictive performance, these models can be considered suitable for toxicity screening of environmental chemicals.

## Introduction

The endocrine system is responsible within the human body for the regulation of many processes such as metabolism, regulation of the internal environment (temperature, water and ions homeostasis), reproduction, growth, and development (Silverthorn et al., 2013). The homeostasis of the endocrine system is a complex mechanism that requires the correct balance of different elements to work properly.

A part of the endocrine system is represented by the hypothalamic-pituitary-thyroid (HPT) axis (Fekete and Lechan, 2014), one of the major neuroendocrine systems of vertebrate organisms, that modulates protein, carbohydrate and fat metabolism (Silverthorn et al., 2013).

Interferences at any level of this system may lead to abnormal functionalities and to adverse effects at various tissue and body levels. In particular, in children, thyroid hormones (THs) are essential for normal growth and development, especially of the nervous system. Because of the importance of these hormones in children, the United States and Canada test all new-borns for thyroid deficiency. Moreover, there is evidence of deficiency in neurodevelopment also in fetal stages linked to maternal hypothyroxinemia (Williams, 2008). In adults, dysregulation of THs brings to pathologies like hyper- and hypothyroidism that have been correlated with adverse outcomes leading to cancer, obesity and type II diabetes mellitus (Pearce, 2012; Wang, 2013). Xenobiotics can interfere with the HPT axis mostly by binding to receptors, enzymes and transporters, thus leading to adverse outcomes such as impaired development, reproduction, neurological function and immune system responses (Danzo, 1997). Given their spread in the contemporary society, endocrine disrupting chemicals represent a major concern in the context of human and environmental health and safety. Indeed, several organizations and entities have developed specific programs and guidelines in order to help countries to design environmental policies and address chemical safety issues. Example of these efforts are the Environmental Health and Safety programme (EHS) of the Organization for Economic Co-operation and Development (OECD) (OECD, 2018), the guidelines provided by the European Food Safety Authority (EFSA) with the technical support of the Joint Research Center (JRC) (Andersson et al., 2018) and the Endocrine Disruptor Screening Program (EDSP) of the United States Environmental Protection Agency (EPA) (EPA, 2017).

Thyroid system disruption can be a consequence of different events including: 1) interference with hypothalamic-pituitary feedback mechanisms; 2) inhibition of thyroperoxidase (TPO); 3) inhibition of glandular iodide uptake by the sodium-iodide symporter; 4) nuclear receptor-mediated increase in the metabolic clearance rate of THs; 5) competitive binding to TH serum binding proteins; 6) inhibition of peripheral iodothyronine deiodinases; 7) interference with the TH receptor complex in target tissues; and/or 8) inhibition of TH transporters in target tissues (Capen, 1994; DeVito et al., 1999; Crofton et al., 2005; Murk et al., 2013). The inhibition of the TPO enzyme, which catalyses many steps of THs synthesis, leads to a reduced synthesis of THs that may in turn lead to a series of dysfunctions related to hypothyroidism, such as reduced basal metabolism and alteration in the lipidic assets. The inhibition of the TPO enzyme can occur as a consequence of prolonged exposure to several chemical compounds and mixtures like pesticides (such as thiocarbamate, thiourea, and triazole) and industrial chemicals (resorcinol, phthalates) (Brucker-Davis, 1998).

In the last decades, the field of toxicology has begun to make use of integrated approaches, including alternative testing methodologies to manage an incredibly large amount of chemicals that could potentially affect environment and human health. Whilst *in vitro* and *in vivo* methods are intended to assess the effects of a substance by administering it directly to a biological system, and are so referred as testing methods, *in silico* approaches rely on the available knowledge about known chemicals, starting from the hypothesis that similar compounds behave in a similar way. Such approaches aim to predict different properties of untested chemicals, such as physico-chemical and structural properties, binding activity to enzymes or receptors, toxicological profile, toxicokinetic properties, and rely on mathematical and statistical methods. They are therefore referred to as non-testing method (NTMs). Examples of NTMs are grouping methods, like read-across and chemical category formation, which are useful for data gap filling; quantitative structure-activity relationship (QSAR) models, used to quantitatively correlate measures of chemical structure to either a physical property or a biological effect (e.g., toxic outcome); physiologically based pharmacokinetic (PBPK) models for predicting absorption, distribution, metabolism and excretion of chemical substances in humans and other animal species (Raunio, 2011).

Computational (*in silico*) methods play an essential role in data generation, data gap filling and chemicals prioritization. In this work, the development, optimization, and evaluation of QSAR models for predicting the capability of xenobiotics to inhibit the TPO enzyme are presented. Experimental TPO *in vitro* inhibition activity data were retrieved from the literature and measured through the Amplex UltraRed-thyroperoxidase (AUR-TPO) assay. Data were curated through various steps, and different classifiers were trained with optimization procedures in order to extract the best predictive models. Variable selection techniques were also considered to improve prediction quality. Performance of the models was evaluated through proper metrics, along with robustness analysis. Models were validated on a completely external set of compounds retrieved from Rosenberg et al. (2017).

## Materials and Methods

### Dataset Preparation

TPO inhibition data were retrieved from Paul-Friedman et al. (2016). Data are related to the Amplex UltraRed-thyroperoxidase (AUR-TPO) assay. Amplex UltraRed (AUR) is a fluorogenic substrate that is converted to Amplex UltrOxRed by horseradish peroxidase in the presence of H_2_O_2_ and serves to detect peroxidase (i.e., TPO) activity (Paul-Friedman et al., 2014). A selectivity (SEL) value was associated to positive hit-calls. AUR-TPO SEL was calculated using the difference between the log IC20 (i.e., the inhibitory concentration at 20%) for the AUR-TPO assay and the log IC20 value of either 1) a luciferase inhibition or 2) a cytotoxicity assay. High IC20 values for either of the two assays account for possible false positive among positive hit-calls. The former assay (luciferase assays) flags for non-specific enzyme inhibition, the latter for cytotoxicity. SEL values were used to stratify active TPO inhibitors in non-selective (NSE) (SEL <0), low selective (LSE) (0 ≤ SEL <1) and high selective (HSE) (SEL ≥1). High selective chemicals were characterized by a larger separation of the AUR-TP assay log IC20 value from confounding activities identified from luciferase and cytotoxicity assays. SMILES were curated by means of a semi-automated in-house procedure described by Gadaleta et al. (2018a). This procedure addresses the identification and the removal of inorganic and organometallic compounds and mixtures, the neutralization of salts, the removal of duplicates (also checking for tautomeric forms) and the standardization of chemical structures. 14 records were removed because it was not possible to identify a unique SMILES; eight inorganic chemicals and 12 that included unusual chemical elements (i.e., those different from H, C, N, O, F, Br, I, Cl, P, S) were removed too. After structure normalization 122 chemicals were identified to have duplicates. For them, a single instance was kept in the dataset with the exception of six duplicates characterized by contradictory experimental category assignment that were removed. The final dataset for the AUR-TPO assay includes 1073 chemicals (751 inactive ones (INA), 105 NSE, 64 LSE and 153 HSE). Three binary datasets were defined by varying the assignment of NSE and LSE compounds to the active and inactive category, while a fourth dataset was built considering only HSE and INA chemicals, while LSE and NSE were discarded. For each dataset, data were randomly split into a training (TrS) and a testing set (TeS) with the train_test_split method of the SciKit-learn’s model selection library [66]. TrS is the set of data that “trains” a model to correctly relate variables (descriptors) to output classes (active/inactive classes). The trained model is then evaluated using it to predict TeS outcomes. In our case, the TrS contained 80% of the data, while the TeS 20%. The inactive/active ratio was kept in each dataset. The four partitioning schemes are summarized in Table 1.

**TABLE 1:** Partitioning schemes. For each dataset, the number of active and inactive chemicals and the related class distribution is reported. For each partitioning scheme, the total number of chemicals (#), the number of active (ACT) and inactive (INA) chemicals, the number of chemicals in the training (TrS) and test set (TeS) and the ratio between inactives and actives is reported.

Dataset (ACT/INA)	INA	ACT	TrS	TeS	INA:ACT	#
HSE/LSE + NSE + INA	920	153	858	215	6:1	1073
HSE + LSE/NSE + INA	866	217	858	215	4:1	1073
HSE + LSE + NSE/INA	751	322	858	215	2.3:1	1073
HSE/INA	751	153	723	181	5:1	904

Additional AUR-TPO assay data were retrieved from Rosenberg et al. (2017). The dataset includes only HSE and INA compounds. Compounds already present in the TrS were removed, then the same curation procedure described for the TrS and the TeS was made. The final dataset includes 631 compounds (538 INA, 93 HSE). The resulting set was used as external set (ES) for the validation of the developed models. The ratio between INA and HSE in the TrS and in the ES is analogous, being 83% in both cases.

**FIGURE 1:**
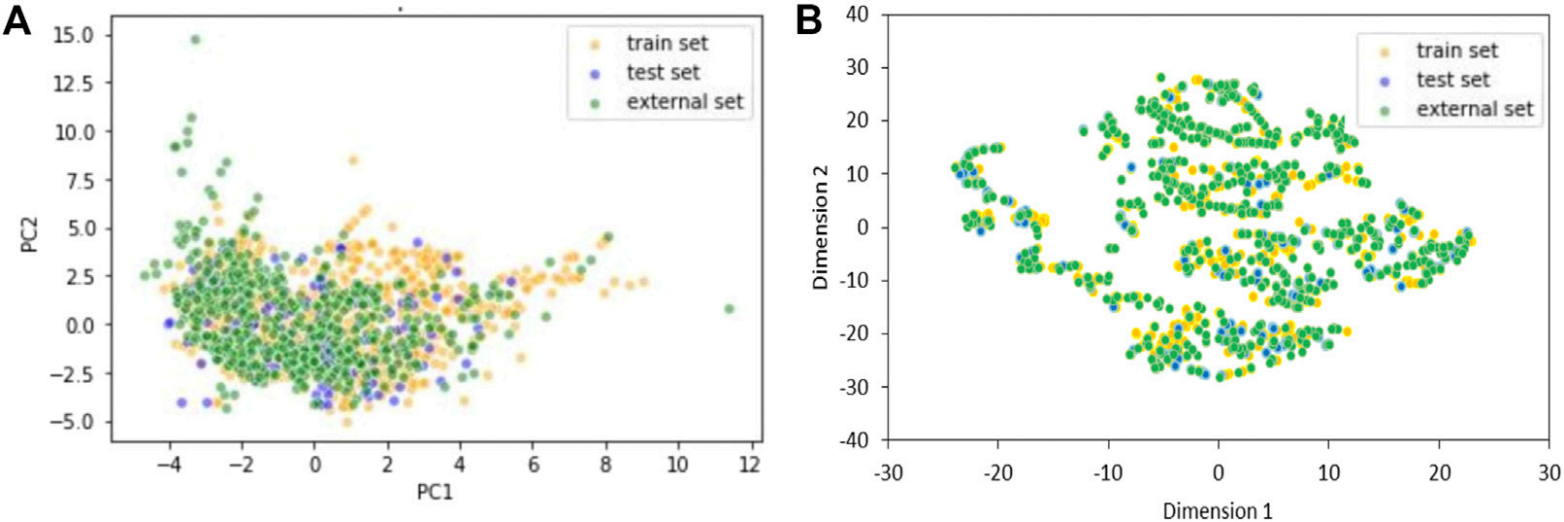
Two components PCA **(A)** and t-SNE **(B)** plots of training, test and external sets including only the 20 selected variables of the KNN model. Yellow dots are TrS chemicals, blue dots are TeS chemicals, and green dots are ES chemicals.

The chemical space covered by the three datasets was further inspected using Principal Component Analysis (PCA) (Figure 1A) and t-distributed stochastic neighbor embedding (t-SNE) (Figure 1B) (theta = 0.5, perplexity = 30) (Van der Maaten and Hinton, 2008).

**FIGURE 2:**
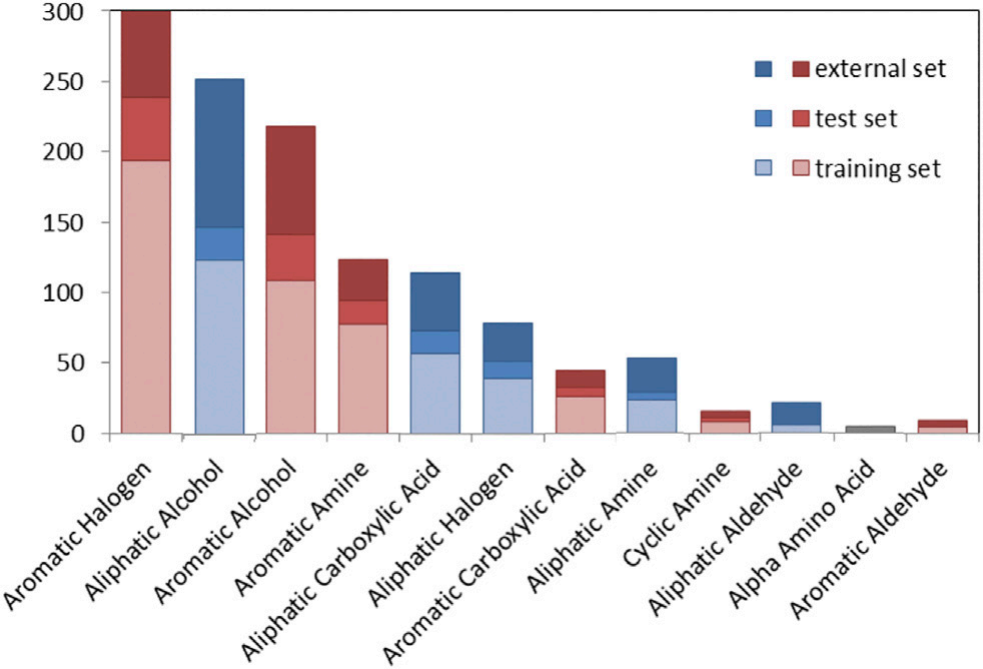
Graphical overview of the number of compounds in the TrS, TeS and ES among chemical classes. Red bars refer to aromatic chemical classes, while blue bars refer to aliphatic classes.

Figure 2 shows the number of chemicals distributed in a series of chemical classes for the TrS, the TeS and the ES. Chemical classes are based on 22 SMARTS codifying functional groups that are included in the RDKit Functional Group Filter KNIME node (https://www.rdkit.org/). Most of the top-represented classes (e.g., aromatic halogens, aromatic alcohols, aliphatic carboxylic acids) likely refer to chemicals that mimic the activity of the natural substrate of the TPO, i.e., the tyrosine residues of thyroglobulin (and their partially iodinated intermediates) (Kessler et al., 2008).

Chemical space analyses confirmed that the TeS is very close to the TrS, as the two datasets were obtained by the same original source of data. Conversely, the external set sometimes covers a chemical space that is slightly different from the one covered by the TrS and the TeS, being a good example of real-life validation.

The complete list of chemicals used for models training and validation are included in the Supporting Information (Supplementary Table S1 for TrS and TeS, Supplementary Table S2 for ES).

### Descriptors Generation and Selection

Molecular descriptors were computed for each chemical in the datasets with DRAGON v7.0.8 software (Kode, 2017). Descriptors having missing values or constant/near-constant variables (i.e., standard deviation <0.01) were removed along with descriptors having an absolute pair correlation higher than 95% with other variables. The final dataset consisted of 619 descriptors. Descriptors values were scaled with a standard normalization (i.e., mean equal to zero and standard deviation equal to 1).

Models were based both on the entire pool of descriptors, and on a reduced pool defined with two variable selection techniques that ranked variables according to a score which indicates their importance. The first variable selection method was based on the relative importance of variables within a Random Forest (Genuer et al., 2010). The importance of a variable is related to its ability to perform a good split on the data, and it is quantified through the Gini impurity score. In the second method, the importance of a feature is evaluated as the decrease of the performance of a classifier when that single feature value is randomly shuffled *n* times. Then, a “baseline” score is compared with the average score of the *n* permutations. The higher the difference between the two scores, the more the feature is considered important (https://scikit-learn.org/stable/modules/permutation_importance.html#id2). In our case, the classifier used was a Random Forest (or Balanced Random Forest) and the balanced accuracy (BA) was used as score. From each of the two lists of ranked variables, the first 20, 30, 50, 100, 120, 130, 150, 160, 170, 200, 250, 300 important features were used to train models and the results obtained from the optimal subset of variables were reported for each classification algorithm. Descriptor selection was performed exclusively on TrS chemicals.

### Modeling Approaches

For each of the partitioning schemes reported in Table 1 and each of the subset of selected descriptors, four classification algorithms, i.e., Balanced Random Forest (BRF) (Breiman, 2001; Dal Pozzolo et al., 2015), Support Vector Machine (SVM) (Vapnik, 1963) K-Nearest Neighbors (KNN) (Altman, 1992) and Random Forest (RF) (Breiman, 2001) were used for modelling. Hyper-parameter tuning was performed in internal validation (10-fold cross validation) in order to identify the optimal set of parameters for each model. The optimized models were then validated on the TeS and performance was compared in order to select a model to evaluate on the ES.

Preliminary modelling attempts performed on the original unbalanced TrS gave poor results (data not shown). As a consequence, in case of RF, SVM and KNN, the original TrS was artificially altered in order to balance the number of active and inactive samples. In particular, the Synthetic Minority Oversampling Technique (SMOTE) was applied to create new synthetic data points to assign to the minority class (Chawla et al., 2002). The TeS was kept unbalanced, in order to evaluate the real capability of classifiers to predict the real-life unbalanced distribution of data. This artificial rebalancing of categories was not applied in the case of BRF, as this algorithm is specifically tailored to handle unbalanced distributions of data through a different resampling (undersampling) within each tree of the samples in majority class (Dal Pozzolo et al., 2015).

**FIGURE 3:**
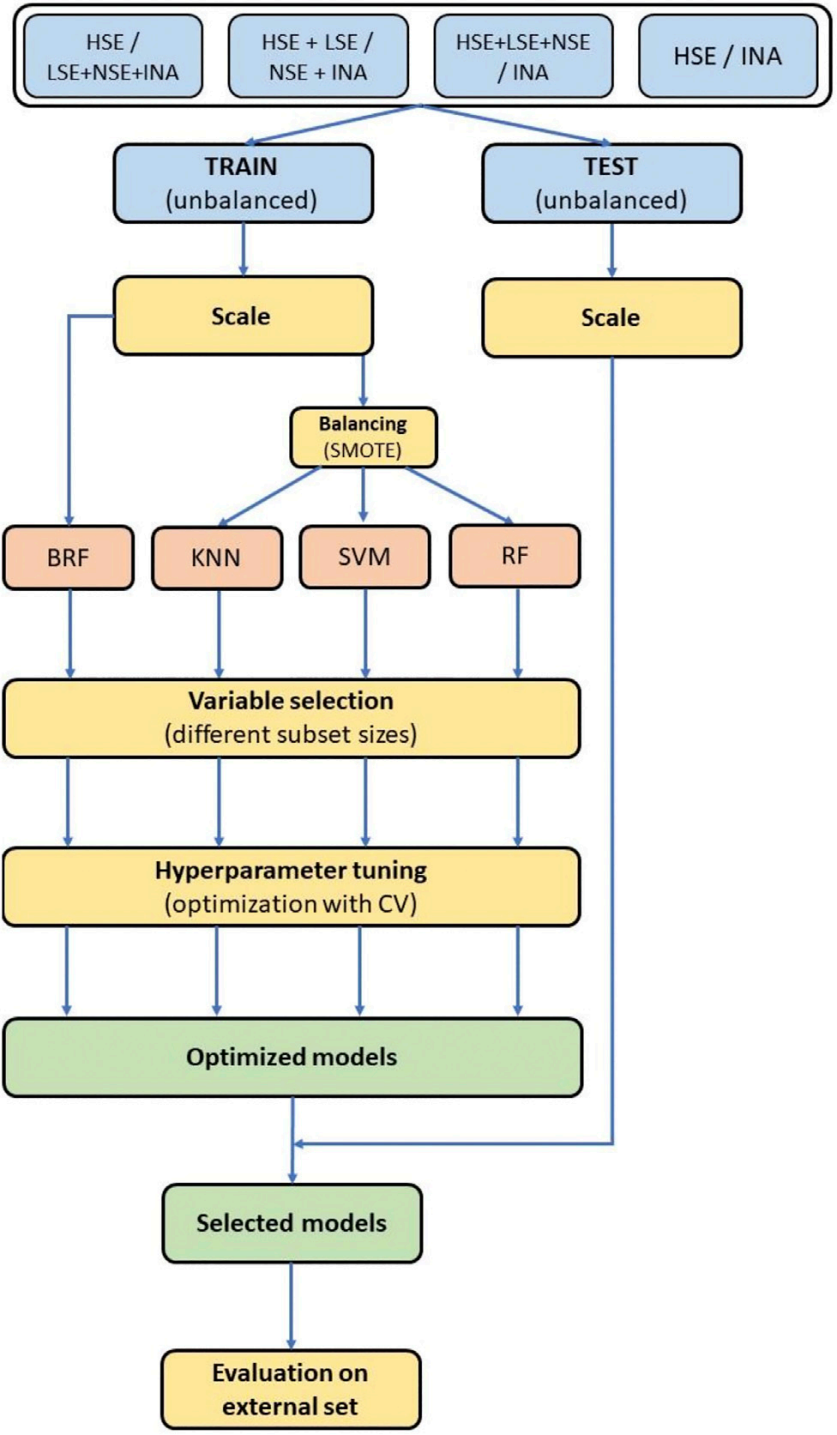
Modelling workflow. Colors of the blocks are related to different phases of the modelling procedure. Light blue blocks: data preparation. Light red block: machine learning. Yellow blocks: data selection and optimization. Green blocks: models selection.

All methods used for data analysis and model development were implemented with Python 3 (van Rossum and Drake, 2003), packages Pandas (McKinney, 2011) for data manipulation and SciKit-learn (Pedregosa et al., 2011) and Imbalanced-learn (Lemaître et al., 2017) for machine learning techniques. An overview of the whole modelling workflow is shown in Figure 3.

In order to refine predictions provided by single models, consensus modelling was applied. In particular, a compound is assigned to a category only when concordant predictions are generated by the top performing models among those developed.

### Performance Evaluation

Cooper’s statistics were used to evaluate the performance of the obtained classifiers (Coper et al., 1979). In particular, Sensitivity (SEN), Specificity (SPE) and BA were calculated as below:

$$SEN=\frac{TP}{TP+FN}$$

$$SPE=\frac{TN}{TN+FP}$$

$$BA=\frac{SEN+SPE}{2}$$

with true positives (TP) and true negatives (TN) being respectively the positive and negative samples correctly classified by the models, while false negatives (FN) and false positives (FP) are respectively the experimentally positive and negative samples that are misclassified.

Matthew correlation coefficient (MCC) (Matthews, 1975) was also calculated, being similarly to BA particularly suitable to evaluate the performance of classifiers on unbalanced distributions of data. MCC ranges from 1 (perfect classification) to -1 (perfect misclassification).

$$MCC=\frac{TP\;TN-FP\;FN}{\sqrt{\left(TP+TN\right)}\left(TP+FN\right)\left(TN+FP\right)\left(TN+FN\right)}$$

Y-scrambling (Kubinyi, 2004; Rücker et al., 2007) was used to evaluate if each of the methods lead to a significant classification. It consists of an iterative random shuffling the activity labels (classes) and a re-training of the model based on shuffled activities. Then, a *p*-value is computed as the percentage of iterations for which the score obtained is greater than the classification score obtained without activity reshuffling, as in the equation below:

$$p-value=\frac{c+1}{n+1}$$

where *c* is the number of iterations giving a score greater than the original, and *n* is the number of times the procedure is repeated. In the present work, 100 permutations were made. For each permutation, BA was computed in cross validation.

## Results

**FIGURE 4:**
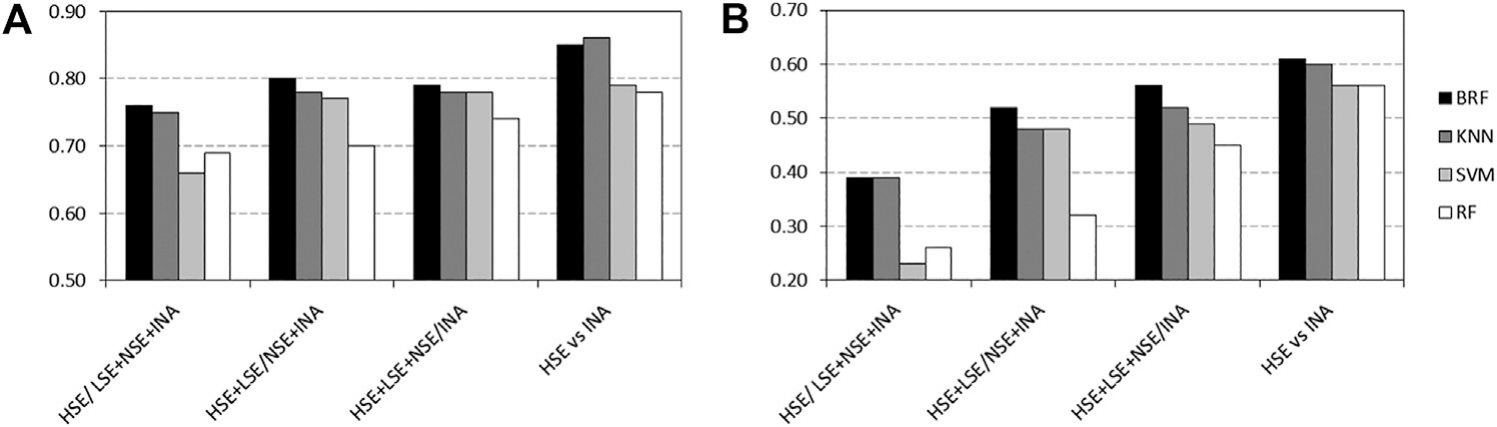
Comparison of **(A)** Balanced Accuracies (BA) and **(B)** Matthews Correlation Coefficients (MCC) values for the top performing TPO classifiers. White bars refer to Random Forests, light grey bars refer to Support Vector Machines, dark grey bars refers to k-Nearest Neighbor while black bars refers to Balanced Random Forests.

Tables 2–5 report performance on the TeS of the best models for each type of classifier and each of the four partitioning schemes described in paragraph 2.1. Figure 4 compares BAs on the TeS of the various models. The comparison of the overall performance, i.e., BA (Figure 4A) and MCC (Figure 4B) of the different classifiers reveals that KNN and BRF were constantly the top performing methods among those considered. BRF reaches BA values in the range of 0.76–0.85 and MCCs in the range of 0.39–0.61 across the various splitting schemes. KNN is quite similar, as it returns BAs in the range of 0.75–0.86 and MCC in the range of 0.48–0.60. SVM are comparable with the former methods in some cases, but they are characterized by a greatest difference between SEN and SPE (Tables 2–5); in particular SVM are characterized by a high false negative rate, i.e., many toxic compounds predicted as safe, that is an issue for the intended real-life use of the models. Conversely, BRF and KNN showed more balanced statistics in the prediction of positive and negative samples. SMOTE-RF is characterized by lower performance with respect of other classifiers.

**TABLE 2:** Performance of the best models developed on the HSE/LSE + NSE + INA partitioning scheme on the TeS. For each model, the following statistics are reported: Balanced Accuracy (BA), Sensitivity (SEN), Specificity (SPE), Matthews Correlation Coefficient (MCC), number of True Negatives (TN), False Positives (FP), True Positives (TP) and False Negatives (FNs).

Classifier	BRF	KNN	SVM	RF
**#Descriptors**	150	30	100	20
**BA**	0.76	0.75	0.66	0.69
**SEN**	0.74	0.71	0.81	0.81
**SPE**	0.77	0.79	0.52	0.57
**MCC**	0.39	0.39	0.23	0.26
**TN**	142	146	95	104
**FP**	42	38	89	80
**TP**	23	22	25	25
**FN**	8	9	6	6
**#**	215	215	215	215

**TABLE 3:** Performance of the best models developed on the HSE + LSE/NSE + INA partitioning scheme on the TeS. For each model, the following statistics are reported: Balanced Accuracy (BA), Sensitivity (SEN), Specificity (SPE), Matthews Correlation Coefficient (MCC), number of True Negatives (TN), False Positives (FP), True Positives (TP) and False Negatives (FNs).

Classifier	BRF	KNN	SVM	RF
**#Descriptors**	170	30	30	130
**BA**	0.80	0.78	0.77	0.70
**SEN**	0.79	0.79	0.70	0.70
**SPE**	0.81	0.77	0.84	0.71
**MCC**	0.52	0.48	0.48	0.32
**TN**	140	133	144	122
**FP**	32	39	28	50
**TP**	34	34	30	30
**FN**	9	9	13	13
**#**	215	215	215	215

**TABLE 4:** Performance of the best models developed on the HSE + LSE + NSE/INA partitioning scheme on the TeS. For each model, the following statistics are reported: Balanced Accuracy (BA), Sensitivity (SEN), Specificity (SPE), Matthews Correlation Coefficient (MCC), number of True Negatives (TN), False Positives (FP), True Positives (TP) and False Negatives (FNs).

Classifier	BRF	KNN	SVM	RF
**#Descriptors**	250	20	30	20
**BA**	0.79	0.78	0.78	0.74
**SEN**	0.77	0.77	0.72	0.63
**SPE**	0.82	0.79	0.83	0.86
**MCC**	0.56	0.52	0.49	0.45
**TN**	124	119	143	147
**FP**	27	32	29	25
**TP**	49	49	31	27
**FN**	15	15	12	16
**#**	215	215	215	215

**TABLE 5:** Performance of the best models developed on the HSE vs INA partitioning scheme on the TeS. For each model, the following statistics are reported: Balanced Accuracy (BA), Sensitivity (SEN), Specificity (SPE), Matthews Correlation Coefficient (MCC), number of True Negatives (TN), False Positives (FP), True Positives (TP) and False Negatives (FNs).

Classifier	BRF	KNN	SVM	RF
**#Descriptors**	160	20	50	20
**BA**	0.85	0.86	0.79	0.78
**SEN**	0.84	0.87	0.68	0.65
**SPE**	0.87	0.85	0.91	0.92
**MCC**	0.61	0.60	0.56	0.56
**TN**	130	127	136	138
**FP**	20	23	14	12
**TP**	26	27	21	20
**FN**	5	4	10	11
**#**	181	181	181	181

Although all classifiers provide satisfactory performance scores for all the partitioning criteria, the inclusion of LSE chemicals in the active category improved the results with respect of the first partitioning scheme (HSE/LSE + NSE + INA), likely due to the reduction of the imbalance between the inactive and active categories. A further improvement of performance is observed in the HSE/INA partitioning scheme obtained after the complete exclusion of low selective positive hit-calls (i.e., LSE and NSE), so the models improved if the dataset contained only the most reliable experimental data.

Y-scrambling was also performed to assess the statistical validity of predictions. In all cases, the average score over 100 permutation is 0.50, confirming that performance obtained is not due to chance. *p*-value of 0.01 indicates that in none of the 100 iterations the “random” score exceeds the actual performance.

Given the results above, the top performing models that are selected for additional validation are the KNN and the BRF models based on the HSE/INA partitioning. The KNN model is based on 20 variables that were selected with the variable importance-based method based on RF, whilst the 160 variables of the BRF model where selected with the permutation-based method using a BRF as estimator.

Internal robustness of the two models was confirmed by performance achieved in 10-fold internal cross validation, with the BRF reaching BA = 0.79 (SEN = 0.74, SPE = 0.83, MCC = 0.49, AUC = 0.85) and the KNN reaching BA = 0.72 (SEN = 0.49, SPE = 0.95, MCC = 0.49, AUC = 0.82).

Table 6 shows the performance of the BRF, KNN and the consensus model on the ES. Performances of the models on the ES confirm the overall good predictivity of the models, being the KNN characterized by a higher SPE, while the BRF shows more balanced statistics. Performances on the ES are in line with the experimental variability reported for the AUR-TPO assay (i.e., BA ∼0.70) (Paul-Friedman et al., 2016). In particular, the BRF returns BA = 0.76 (BA = 0.85 on the TeS) while the KNN has BA = 0.78 (BA = 0.86 on the TeS).

**TABLE 6:** Comparison of the performance of the BRF and KNN, and ensemble models based on the HSE/INA splitting schemes on the ES. For each model, the following statistics are reported: Balanced Accuracy (BA), Sensitivity (SEN), Specificity (SPE), Matthews Correlation Coefficient (MCC), number of True Negatives (TN), False Positives (FP), True Positives (TP) and False Negatives (FNs).

Classifier	BRF	KNN	Consensus
**#Descriptors**	160	20	-
**BA**	0.76	0.78	0.82
**SEN**	0.72	0.68	0.75
**SPE**	0.79	0.88	0.89
**MCC**	0.40	0.49	0.56
**TN**	427	472	411
**FP**	111	66	50
**TP**	67	63	55
**FN**	26	30	18
**#**	631	631	534

**FIGURE 5:**
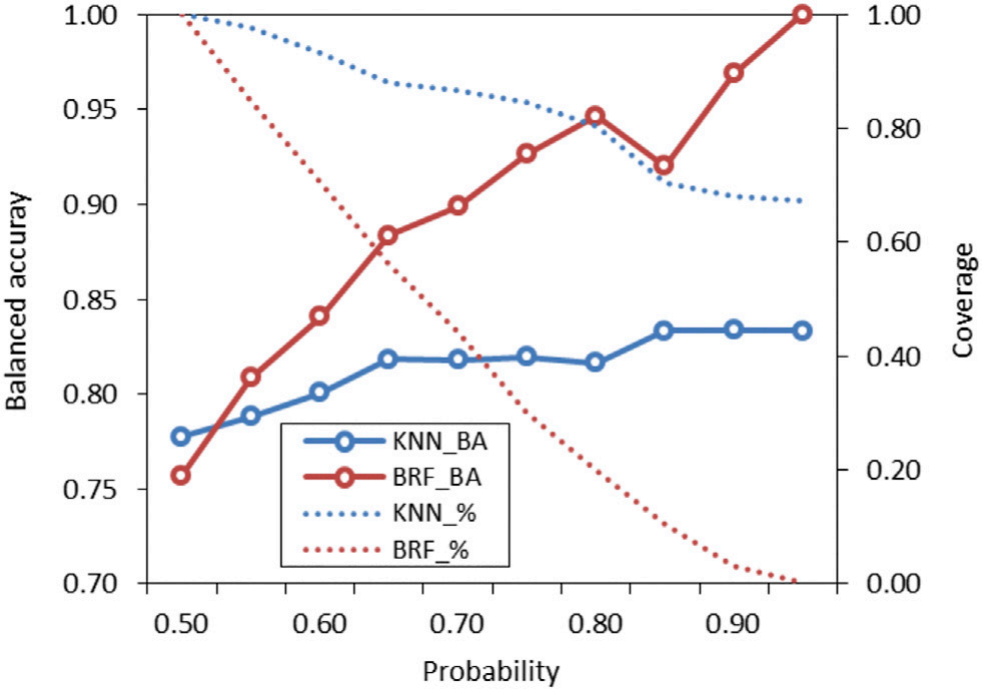
Variation of performance of the KNN and the BRF models on the ES based on probability threshold for prediction domain definition. Solid lines refer to the overall BA while dotted lines refer to the percentage of compounds included in the domain of the model (coverage) on the total included in the ES.

The probabilities associated to predictions were used to identify predictions falling within the response domains of the models (Gadaleta et al., 2016). Probabilities reflect how the chemical space around the target is covered by the model, as higher probabilities are associated to predictions when the model has been adequately trained with training samples that are structurally close to the target. Conversely, low probabilities indicate that few compounds are in the TrS with structural features similar to the target. These predictions are considered less reliable and potentially outside of the model’s domain. Figure 5 shows how the performance (BA) of the KNN and the BRF models and the percentage of predicted compound (coverage) of the ES vary when predictions with lower probabilities are discarded. In both cases, the increase of the probability threshold is associated to an increase of the overall performance and to a decrease of the percentage of compounds that are predicted, with a trend that is more marked for the KNN model with respect of the BRF. The use of different thresholds acknowledges the “fuzzy” nature of the applicability domain and the need of defining confidence limits instead of a simple “in or out” classification (Jaworska et al., 2005). Considering the need to find a reasonable compromise between predictive performance and coverage of the models, the authors suggest that, for the BRF, external predictions with associated probabilities higher than 0.65–0.75 may be considered inside the applicability domain of the model, while for the KNN model a threshold of 0.60–0.65 is suggested.

Consensus modelling was applied by integrating results of the kNN and the BRF models. 534 out of 631 compounds (about 85%) have a concordant prediction while, of the 97 remaining compounds, 69 are predicted correctly by the KNN and 28 by the RBF model respectively. Overall, the consensus model improved the performance of the single models (BA = 0.82) at the expense of a slightly loss of predictions (about the 15% of the whole ES) (Table 6).

## Discussion

In this work, the development and the validation of QSAR models for predicting the inhibition activity of xenobiotics on the TPO enzyme is presented. Various machine learning techniques and schemes for data selection/partition were proposed to develop models, that were systematically optimized and evaluated for their statistical robustness and predictivity through internal and external validation. Two top-performing models based on KNN and BRF algorithms were identified based on their external predictivity evaluated on the TeS, then additional validations were made on the selected models through the exploitation of a completely external dataset. This additional validation was performed on data that were independent from those used for model development and testing. Validation results observed on the ES, although cannot be generalized to the whole chemical universe, are a good simulation of a real-life hazard evaluation made on new chemicals, and confirmed the good predictive performance observed for the TrS and the TeS.

Another important point solved by the external validation is that it allows to check for the presence of overfitting within the model. Indeed, one of the main pitfalls related to the use of a single training-test split is that performance observed for the test set may be biased and dependent on the seed used for the split (i.e., different and possible worse validation performance may be observed for a different training-test split). Conforting performance observed on ES data (that have been retrieved from a different and independent sources with respect of TrS and TeS data) may allow to reduce the doubt of being in presence of overfitting, as performance on external data proved to be good and comparable with the experimental variability.

In both cases, the top-predictive models were based on a dataset that excludes NSE and LSE samples, and includes HSE data as the only representative of the “active” category. This is not unexpected; indeed these models are based on a dataset that excludes the chemicals characterized by an ambiguous categorization due to confounding activities (LSE and NSE hit-calls) (see paragraph 2.1) and includes only the most reliable experimental data.

At the best of our knowledge, this is one of the few attempts available in the literature to model TPO inhibitory activity. Another QSAR study was proposed by Rosemberg et al. (2017) that applied partial logistic regression (PLR) implemented in Ledscope^®^ Predictive Data Miner (Leadscope, Inc, 2016) to develop models for the prediction of AUR assay data. The same categories of compounds (HSE/INA) were considered for model development. The study reported an external BA equal to 0.85 that is analogous to the accuracy values observed on the TeS for the BRF and KNN models presented here, but the performance of the PLR model is associated a much lower prediction coverage (i.e., 54.5% for the PLR model). Recently Garcia de Lomana et al. (2020), proposed a set of models predicting the interference of small molecules with nine targets involved in the thyroid hormone homeostasis (deiodinase 1, 2 and 3, transtiretrin, TPO, thyroid releasing hormone receptor and thyroid stimulating hormone receptor). Models were developed using five machine learning algorithms in combination with three data balancing approaches. In addition, multi-task models were also explored. The best model reported for TPO prediction was an oversampling-based XGBoost (XGB) that was developed from a combination of ToxCast data and data from Paul-Friedman et al. (2016). The XGB showed the following performance in 10-fold cross-validation: F1 = 0.83, MCC = 0.67, BA = 0.82, AUC = 0.90.

Both of the KNN and the BRF models presented here are developed with the aim of handling the unbalance towards negative samples that is typical of the majority of the distributions of biological data, such as the inhibition activity on the TPO enzymes here. In the case of the KNN, this aspect is handled by artificially rebalancing the initial distribution of data through a SMOTE technique. In the case of the BRF, no data pre-treatment is needed, as this method automatically addresses the unbalance between categories that may exist in a training set, through a balanced resampling of data used to train each decision tree in the BRF. Results obtained here confirmed our previous experiences on the suitability of this technique to model unbalanced distributions of data (Gadaleta et al., 2018b; 2019).

**FIGURE 6:**
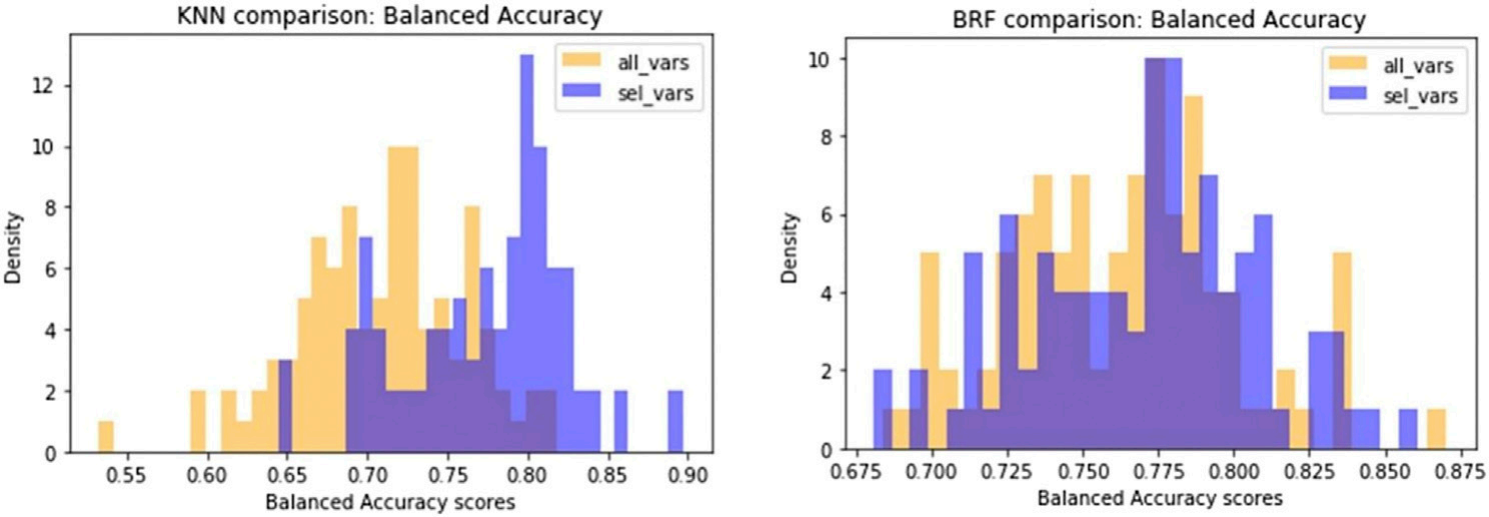
Comparison of performance of models developed on random subsets of chemicals extracted from the ES. Violet bars refer to the distribution of BA of models based on the selected optimal features, while yellow bars are the distribution of BA of models based on the entire pool of descriptors.

**FIGURE 7:**
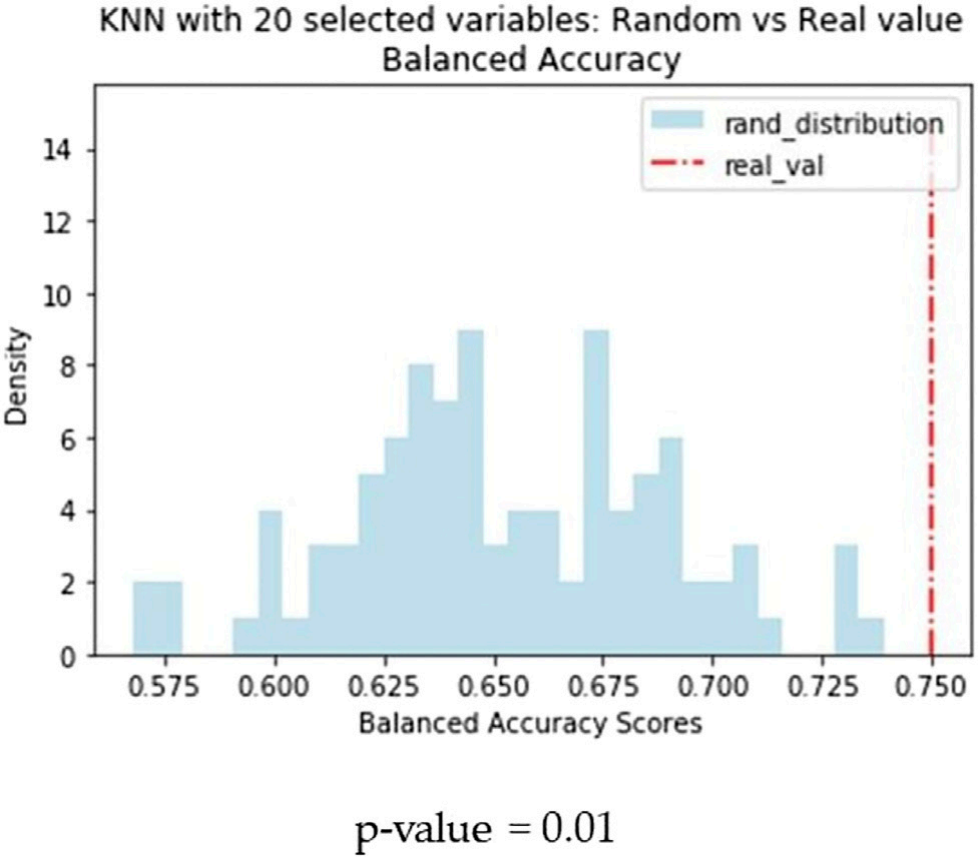
Comparison of performance of the KNN models based on random subsets of features. Blue bars refer to the distribution of BA of models based on random subsets of 20 features, the red dashed line indicates the performance of the KNN model based on the 20 optimal features selected through the RF-based feature importance ranking.

Another difference observed between the two models is that KNN returns the best results when trained on a small subset of optimal molecular descriptors (20) identified by means of variable selection techniques. Conversely, a larger pool of variables (160) are needed to optimise the performance of the BRF model. In order to confirm the different relevance of the feature selection procedure for the two methods, we made an iterative random selection of 200 compounds from the ES that were used to validate models based on different sets of variables. 100 iterations were made. Figure 6A compares the results obtained in the iterations using the entire pool of variables or the best selected subset for the specific technique (20 for KNN and 160 for RBF). It shows that in case of BRF, the models developed with and without variable selection have similar performance (BA) when applied to the ES subsets. This confirms one of the RF algorithm’s specific strengths, i.e., its own implicit feature selection, as it is intrinsically able to identify the most relevant variables and is less sensitive to noisy ones (Anger et al., 2014). A different picture is shown for the KNN model (Figure 6B), that presents increased performance when associated to a reduced pool of selected variables. This means that KNN method is more sensitive to noisy variables than BRF, and that a previous feature selection can help in reaching higher performance. In both cases, the use of a reduced pool of variables may provide advantages in terms of simplicity and interpretability. The validity of the feature selection method (variable importance-based feature ranking) that returned the 20 optimal variables used for the KNN development was further confirmed by comparing the performance of the KNN models with a series of 100 models developed on random subsets of 20 variables selected among the entire pool of descriptors. As shown in Figure 7, none of the iterations reached performance comparable with the KNN model here considered, confirming that the method used was able to identify and remove irrelevant variables.

Table 7 reports the list of the top 20 high ranked variables of the KNN model. Some of them refer to the presence of chemical groups that resemble those included in the natural substrates of TPO, i.e., the tyrosine residues present in the thyroglobulin proteins that are subjected to a series of iodination steps (Kessler et al., 2008). C-026 is an atom-centred fragment codifying for the presence of the R--CX—R group, where -- is an aromatic bond and X is an heteroatom. Similarly to nArOH (number of aromatic hydroxyls), it is likely to codify for the presence of aromatic hydroxyls that mimic the structure of the tyrosine side chain. Analogously, nCb- (number of substituted benzenes), nCbH (number of unsubstituted benzenes) and Uc (unsaturation count, that checks for the presence of double and triple bonds), account for the presence of aromatic moieties resembling the structure of the tyrosine. The number of halogen atoms, checked by the nX descriptors, is also relevant as partially iodinated intermediates (e.g., 3-iodotyrosine) are natural substrates of the enzyme. CATS_02_DL and CATS_03_DL account for the presence of potential pharmacophore points at a given topological distance. In both cases, the pharmacophore points are a lipophilic feature (e.g., a halogen, or one of the carbons of the aromatic residue) and a hydrogen-bond donor, that is likely represented by the hydroxyl group of the tyrosine natural substrate. MLOGP is a general estimation of the lipophilicity of the molecule, and here is likely accounting for hydrophobic non-binding interactions that are established between the aromatic substrates and the residues present in the active site of the TPO enzyme.

**TABLE 7:** List of the top-20 high ranked variables that are included in the KNN model.

GATS1e	MATS1p
NArOH	nCb-
CATS2D_02_DL	NX
MATS1e	Uc
MATS1s	'P_VSA_i_1'
C-026	'SpMAD_B(v)
CATS2D_03_DL	NCbH
B10 [C-C]	GATS1s
MATS1m	MLOGP
'SpMax2_Bh(s)	Eta_C_A'

Integrated modeling was also applied in order to improve predictions of single models. As showed here and previously in the literature (Tropsha, 2010; Gadaleta et al., 2017; Benfenati et al., 2019; Gadaleta et al., 2019) integrated modeling often allows to reach the highest external prediction power (BA = 0.82) compared to any individual model used in the integrated prediction (BA = 0.76–0.78), even with the application of a straightforward strategy such as unanimity. This is particularly true when the individual models have been developed using different approaches and modeling techniques, such as in this specific case. In this regard, the improved performance of the integrated method can be explained by the fact that it compensates for and corrects the limitations of individual techniques.

**FIGURE 8:**
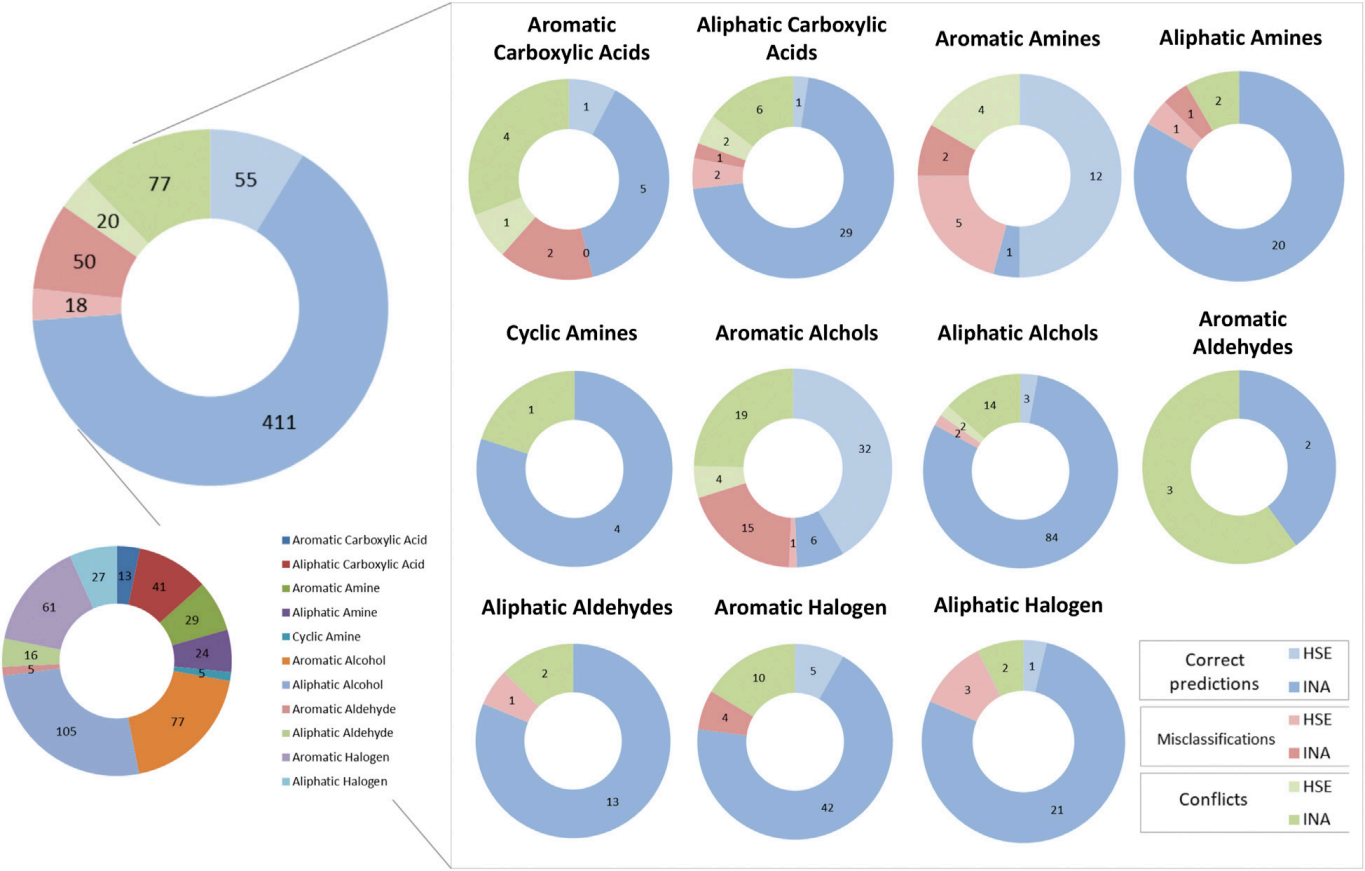
Graphical representation of the number of concordant predictions, misclassifications and conflicts between the KNN and the BRF models across the various chemical classes of the ES. Blue slices of the pie charts indicate concordant correct predictions, Red slices indicate concordant misclassifications, while green slices indicate conflicts between the two models. Light-colored slices refer to experimentally HSE compounds, while dark-colored slices refer to experimentally INA compounds.

Figure 8 depicts a comparison between predictions made by the KNN and the BRF model to identify correct predictions and misclassifications in common between the two models and conflicts across the chemical categories defined as in paragraph 3.1. Overall, common correct predictions are generated for the 77% of ES compounds. Common misclassifications represent the 11% of the ES, while conflicts between the two models are the 15%. In the latter cases, the relative abundance of HSE samples that are misclassified (26% of the misclassifications) or that are conflictual (21% of all the conflicts) is slightly higher than the one observed for correct prediction (12% are HSE, the 88% are INA). This is likely due to the unbalance of the training set towards INA samples, that make the models slightly more accurate when predicting negative chemicals, despite the use of methods to rebalance active and inactive samples minimized this behaviour. The majority of the chemical categories had similar behaviours with respect of the whole dataset. Some categories have a higher relative percentage of conflicts, e.g., aromatic carboxylic acids (38%) and aromatic aldehydes (60%) that are likely due to the low representativeness of the chemical classes in the TrS of the models. Conversely, some classes have slightly higher numbers of common misclassifications, e.g., aromatic amines and aromatic alcohols (21% of the predictions for both the classes). A curious behaviour was observed for the aromatic alcohols. Common correct predictions represent only the 49% of all the predictions made for chemicals in this class, while a relatively high percentage of common misclassifications (21%) and conflicts (30%) was observed. Moreover, aromatic alcohols are nearly evenly distributed in the ES (48% HSE, 52% INA), however common correct predictions are nearly always HSE (84% of correct predictions), while a big percentage of common misclassifications (94%) and conflicts (83%) are experimentally INA. This may indicate a tendency of the model to overestimate the toxicity of aromatic alcohols.

## Conclusion

Predictive toxicology is assuming a high relevance in chemical hazard and risk assessment procedures thanks to the recent advances in machine leaning methods and computational power. Encouraged by these advancements, we proposed in the present study a procedure that led to the development of two QSAR models based on AUR assay data to predict the inhibitory potential of chemicals for TPO. These two QSAR models, based on BRF and KNN respectively, show good external predictivity (BA = 0.76–0.78 on the ES) that is in line with reported variability of the *in vitro* AUR-TPO assay (BA ∼0.70) (Paul-Friedman et al., 2016) and can be considered, for this reason, useful as resources for hazard assessment of large quantities of chemicals to which the population is exposed every day.

QSARs and, more in general, NTMs can be also useful to reveal mechanistic properties not commonly investigated at the whole organism level, to generate new hypothesis and to design more refined testing strategies, also towards animal testing reduction. On the other hand, testing strategies are essential in order to validate newly produced data and hypothesis and of course to generate, in turn, new data that can be used to refine *in silico* predictive models.

The authors propose the models here as part of Integrated Testing Strategies (ITS), with *in silico* methods acting as a first tier to screen large quantitative of chemicals in a short time and to give indication to higher tier testing methods (e.g., *in vitro*) for a refinement of the preliminary results returned by computational models. In particular, the predictions of the models presented here can be used to reduce the number of the chemicals needing confirmatory *in vitro* testing only to those characterized by predictions with low reliability, with a sensible save in terms of money and time needed to perform the *in vitro* assays. Moreover, the models show variabilities that are similar to the assay, and consequently they may be used as a replacement of the AUR-TPO *in vitro* test when this is technically impossible to perform, e.g., for very large numbers of chemicals and/or when laboratory resources are not available. When multiple methods (e.g., *in silico* and *in vitro*) methods are available, a weight of evidence approach is recommended, according to which all data and information generated from all the strategies should be taken into consideration when assessing the desired property of a chemical, weighting each evidence according to its relevance and reliability, as proposed by the EFSA guidance on the matter (Hardy et al., 2017).

The QSARs models presented here are freely available and have been implemented in the freeware KNIME Analytics Platform (v. 4.3.3) (Berthold et al., 2008), making them immediately available for use for scientists and regulators.

## Data Availability

The original contributions presented in the study are included in the article/Supplementary Material, further inquiries can be directed to the corresponding author. The KNIME implementations of models are available at https://github.com/DGadaleta88/TPO_QSAR.
